# Isolation and Propagation of Laboratory Strains and a Novel Flea-Derived Field Strain of *Wolbachia* in Tick Cell Lines

**DOI:** 10.3390/microorganisms8070988

**Published:** 2020-07-01

**Authors:** Jing Jing Khoo, Timothy J. Kurtti, Nurul Aini Husin, Alexandra Beliavskaia, Fang Shiang Lim, Mulya Mustika Sari Zulkifli, Alaa M. Al-Khafaji, Catherine Hartley, Alistair C. Darby, Grant L. Hughes, Sazaly AbuBakar, Benjamin L. Makepeace, Lesley Bell-Sakyi

**Affiliations:** 1 Tropical Infectious Diseases Research and Education Centre, Level 2, High Impact Research Building, University of Malaya, Kuala Lumpur 50603, Malaysia; jing.khoo@um.edu.my (J.J.K.); nurulainihusin@yahoo.com (N.A.H.); limfs92@gmail.com (F.S.L.); mulyamustika@um.edu.my (M.M.S.Z.); sazaly@um.edu.my (S.A.); 2 Department of Entomology, University of Minnesota, 219 Hodson Hall, 1980 Folwell Avenue, Saint Paul, MN 55108, USA; kurtt001@umn.edu; 3 Department of Infection Biology and Microbiome, Institute of Infection, Veterinary and Ecological Sciences, University of Liverpool, 146 Brownlow Hill, Liverpool L3 5RF, UK; alexbel@liverpool.ac.uk (A.B.); csguy@liverpool.ac.uk (C.H.); acdarby@liverpool.ac.uk (A.C.D.); blm1@liverpool.ac.uk (B.L.M.); 4 College of Veterinary Medicine, University of Al-Qadisiyah, Qadisiyah 54004, Iraq; supervisore2@yahoo.com; 5 Departments of Vector Biology and Tropical Disease Biology, Centre for Neglected Tropical Disease, Liverpool School of Tropical Medicine, Pembroke Place, Liverpool L3 5QA, UK; Grant.Hughes@lstmed.ac.uk

**Keywords:** *Wolbachia*, tick cell line, *Ctenocephalides*, flea, Malaysia, in vitro culture, phylogeny

## Abstract

*Wolbachia* are intracellular endosymbionts of several invertebrate taxa, including insects and nematodes. Although *Wolbachia* DNA has been detected in ticks, its presence is generally associated with parasitism by insects. To determine whether or not *Wolbachia* can infect and grow in tick cells, cell lines from three tick species, *Ixodes scapularis*, *Ixodes ricinus* and *Rhipicephalus microplus*, were inoculated with *Wolbachia* strains *w*Stri and *w*AlbB isolated from mosquito cell lines. Homogenates prepared from fleas collected from cats in Malaysia were inoculated into an *I. scapularis* cell line. Bacterial growth and identity were monitored by microscopy and PCR amplification and sequencing of fragments of *Wolbachia* genes. The *w*Stri strain infected *Ixodes* spp. cells and was maintained through 29 passages. The *w*AlbB strain successfully infected *Ixodes* spp. and *R. microplus* cells and was maintained through 2–5 passages. A novel strain of *Wolbachia* belonging to the supergroup F, designated *w*CfeF, was isolated in *I. scapularis* cells from a pool of *Ctenocephalides* sp. cat fleas and maintained in vitro through two passages over nine months. This is the first confirmed isolation of a *Wolbachia* strain from a flea and the first isolation of any *Wolbachia* strain outside the “pandemic” A and B supergroups. The study demonstrates that tick cells can host multiple *Wolbachia* strains, and can be added to panels of insect cell lines to improve success rates in isolation of field strains of *Wolbachia*.

## 1. Introduction

*Wolbachia* is a genus of obligate intracellular endosymbiotic gram-negative bacteria of the family Anaplasmataceae in the order Rickettsiales. *Wolbachia* infect two phyla in the Ecdysozoa: the Arthropoda and the Nematoda, with a much broader range of host species in the former than in the latter. Although only one species, *Wolbachia pipientis*, has been formally described [1], the genus has been separated by multi-locus sequence typing (MLST) into ~18 clades or “supergroups” [2,3,4]. Core genome alignments for supergroups suggest that they can be considered at least equivalent to species rank, with some containing sufficient diversity for more than one species [5]. *Wolbachia* is best known for its ability to induce five distinct reproductive manipulations in arthropod hosts (cytoplasmic incompatibility (CI), induction of parthenogenesis, male killing, feminisation and meiotic drive), all of which favour its spread by reducing resource competition from males (a dead-end host) or imposing a fitness cost on uninfected females [6,7,8,9]. However, these parasitic phenotypes appear to be largely confined to the “pandemic” supergroups A and B that infect ~50% of terrestrial arthropod species [10,11]. In other cases, *Wolbachia* form obligate and putatively beneficial relationships with their hosts, including strains from supergroups C and D in nematodes and E in springtails [12,13].

*Wolbachia* is found in most of the major groups of haematophagous arthropods, including biting Diptera and Hemiptera, fleas, lice and parasitic mites [14,15,16,17,18,19,20,21,22,23,24,25,26]. The CI phenotype, in which the progeny of crosses between infected males and uninfected females (or females carrying an incompatible *Wolbachia* strain) die early in development, is common in blood-feeding Diptera [21,25,27]. In contrast, a supergroup F *Wolbachia* strain in bedbugs is a nutritional mutualist, providing B vitamins for its host that are deficient in the blood meal [18,20]. *Wolbachia* has long been of applied interest for disease control, as release of *Wolbachia*-infected male pest insects can suppress natural populations where the females are uninfected or harbour an incompatible strain [28]. In filarial nematodes that cause neglected tropical diseases such as onchocerciasis and lymphatic filariasis, elimination of *Wolbachia* using antibiotics can safely clear adult worm infections, unlike conventional anthelmintics [29]. Finally, *Wolbachia* infections can suppress the dissemination and transmission of pathogens in insects, especially when transinfected into a novel host [30]. This phenomenon is the basis for several control programmes releasing *Wolbachia*-infected *Aedes aegypti* to reduce the transmission of dengue and other arboviruses [31].

The order Ixodida is the only large group of haematophagous arthropods in which the status of *Wolbachia* infections still remains ambiguous. While many studies have reported the presence of *Wolbachia* in ticks using molecular methods [32,33,34,35,36,37,38,39,40,41,42,43,44,45], it is unclear whether ticks are themselves infected with *Wolbachia*, or if the bacteria are present within cells of species of the parasitic wasp genus *Ixodiphagus* [46] or other parasites of ticks such as nematodes [39] or mites. Recent studies have yielded strong indications that the latter scenario may be the correct one, as *Wolbachia*-positive *Ixodes ricinus* ticks are almost always positive for *Ixodiphagus* DNA [47,48].

However, the question remains whether or not tick cells are capable of supporting infection and growth of *Wolbachia*. To answer this, we first tested the ability of cell lines with known broad susceptibility to infection with intracellular bacteria, derived from *I. ricinus*, *Ixodes scapularis* and *Rhipicephalus microplus*, to support the replication of two laboratory strains of *Wolbachia* derived from different insect hosts. We then applied an *I. scapularis* cell line in an attempt to isolate *Wolbachia* or other intracellular bacteria from field-collected fleas in Malaysia.

## 2. Materials and Methods

### 2.1. Tick Cell Lines

The *I. scapularis* cell lines ISE6 [49] and ISE18 [50] and the *I. ricinus* cell line IRE11 [51] were maintained at 28 °C or 32 °C in L-15C300 medium supplemented with 10% tryptose phosphate broth (TPB), 10% foetal bovine serum (FBS) and 0.1% bovine lipoprotein (MP Biomedicals, Solon, OH, USA) [52]. The *I. scapularis* cell line IDE8 [50] was maintained in flat-sided culture tubes (Nunc, Thermo Fisher, Loughborough, UK) at 32 °C in L-15B medium [53] supplemented with 10% TPB, 10% FBS, 0.1% bovine lipoprotein, 2 mM L-glutamine and antibiotics (100 units/mL penicillin and 100 µg/mL streptomycin). The *R. microplus* cell line BME/CTVM23 [54] was maintained in flat-sided culture tubes at 28 °C in L-15 (Leibovitz) medium supplemented with 10% TPB, 20% FBS, 2 mM L-glutamine and antibiotics. All cell lines were maintained with weekly medium change and subculture at 1–3 monthly intervals.

### 2.2. Wolbachia Strains

For experiments with *Ixodes* spp. cell lines, the *w*Stri strain, originally isolated from the small brown planthopper *Laodelphax striatellus* into the *Aedes albopictus* cell line NIAS-AeAl-2 [55] and kindly provided by H. Noda, National Institute of Agrobiological Sciences, Tsukuba, Japan [56], was propagated in AeAl-2 cells maintained in L-15C300 supplemented as above. The *w*AlbB strain, originally isolated from the mosquito *Ae. albopictus* into the *Ae. albopictus* cell line Aa23 [57] and kindly provided by S.L. Dobson, University of Kentucky, was propagated in Aa23 cells also maintained in supplemented L-15C300 medium. For experiments with the *R. microplus* cell line BME/CTVM23, the *w*AlbB strain, originally provided by Scott O’Neill, Yale University School of Medicine, was transferred from Aa23 cells to *Ae. albopictus* C6/36 cells [58] maintained in a 1:1 mixture of Schneider’s modified *Drosophila* medium (Merck, Sigma Aldrich, Gillingham, UK) and Mitsuhashi and Maramorosch medium (Geneflow Custom Media, Geneflow, Lichfield, UK) supplemented with 10% FBS and 2 mM L-glutamine. Infected mosquito cell cultures were maintained at 28 °C with weekly medium change and occasional subculture.

### 2.3. Preparation of Cell-Free Wolbachia Suspensions and Inoculation of Tick Cell Lines

For experiments with wAlbB and *w*Stri in *Ixodes* spp. cell lines, cell-free *Wolbachia* were initially used to inoculate tick cell cultures. *Wolbachia* were released from heavily infected *Ae. albopictus* cells by forcibly passing infected cell suspensions through a 25 G needle. The resultant suspension was filtered through a 2 µm syringe filter and inoculated into tick cell cultures. To enhance infection rates, filtered *Wolbachia* were transferred to a 2 mL microfuge tube containing tick cells (1–2 × 10^6^ cells/mL in 1.5 mL) centrifuged at 5000× *g* for 5 min and allowed to sit at room temperature for 30 min prior to seeding into culture flasks [59]. Cultures were incubated at 28 °C (*w*AlbB) or 32 °C (*w*Stri). Once established in tick cell lines, *Wolbachia* were maintained by inoculating uninfected tick cell cultures with a suspension of infected cells at dilutions of 1:5 for *w*AlbB and 1:10–1:20 for *w*Stri.

For experiments with *w*AlbB in *R. microplus* cells, a 1.2 mL aliquot of resuspended infected C6/36 cells was diluted with a further 0.5 mL Schneider’s modified *Drosophila* medium, passed ten times through a bent 26-gauge needle and centrifuged at 1500× *g* for 5 min. A 0.3 mL aliquot of the supernate was added to a 2.2 mL culture of BME/CTVM23 cells in a flat-sided culture tube, and to an uninfected culture of C6/36 cells in L-15 (Leibovitz) medium supplemented as above but with 10% FBS, in a flat-sided tube. Cultures were incubated at 28 °C; once established, *Wolbachia* were subcultured by passaging resuspended infected BME/CTVM23 onto fresh cells at a dilution of ~1:10.

### 2.4. Preparation of Homogenate from Field-Collected Fleas

*Ctenocephalides* sp. fleas were collected from domestic cats from a village of indigenous people, also known as the Orang Asli, in Perak, Malaysia (4°18′53″ N, 100°55′49″ E). All field sampling was conducted with the approval of the University of Malaya Institutional Animal Care and Use Committee as well as the Department of Orang Asli Development in Malaysia. Live fleas were immobilised at −80 °C for 15 min and identified to genus level using morphological keys [60], followed by surface decontamination by immersing the fleas into 0.1% benzalkonium chloride solution for 5 min. The fleas were then rinsed with 70% ethanol followed by sterile water and allowed to dry on a piece of sterile filter paper. The flea exoskeleton was cut open with a sterile needle to separate the internal organs from the exoskeleton. Organs from five individual fleas were pooled and transferred into flat-sided culture tubes previously seeded with IDE8 cells in complete L-15B medium containing antibiotics. The combined cell and organ cultures were maintained at 28 °C with weekly medium change (3/4 volume). Once bacterial infection was established, subculture was performed by transferring 0.2 mL of supernate from the infected culture to a fresh tube of IDE8 cells.

### 2.5. Examination of Wolbachia-infected Cultures by Microscopy

Live infected tick cell cultures were monitored by weekly inverted microscope examination. At intervals of 1–7 weeks post inoculation (p.i.) for cultures inoculated with *w*AlbB and *w*Stri, and at 8 months p.i. for cultures inoculated with flea organs, tick cells were resuspended by pipetting and cytocentrifuge smears were prepared from small aliquots (~50 µL) of cell suspension, stained with Giemsa and examined at ×500–1000 magnification. *Wolbachia*-infected BME/CTVM23 cells were prepared for transmission electron microscopy as described previously [61].

### 2.6. Molecular Confirmation of Wolbachia and Host Cell Identity

At intervals, 200–500 µL samples of whole culture suspension were collected and DNA was extracted using commercial kits (Qiagen, Germantown, MD, USA; Qiagen, Manchester, UK; Macherey-Nagel, Düren, Germany) following the manufacturers’ instructions. Standard PCR amplification of fragments of the pan-bacterial 16S rRNA sequence and *Wolbachia wsp*, *coxA*, *fbpA*, *ftsZ*, *hcpA* and *gatB* genes was carried out according to published protocols [3,33]. All standard PCR amplifications included a negative control (water) and appropriate positive controls: DNA extracted from *w*Stri-infected NIAS-AeAl-2 cells, *w*AlbB-infected Aa23 or C6/36 cells for *Wolbachia*-specific PCRs or *Rickettsia raoultii*-infected BME/CTVM23 cells [62] for the pan-bacterial 16S rRNA PCR. PCR products were visualised by agarose gel electrophoresis, and positive PCR products were purified using commercial kits (Qiagen, Germantown, MD, USA; New England Biolabs, Hitchin, UK; Macherey-Nagel, Düren, Germany) and sequenced from both ends using a Sanger sequencing service. To confirm the identity of the bacteria in *w*AlbB-infected BME/CTVM23 cells and the absence of contaminating mosquito cells, qPCR amplification of fragments of the *Wolbachia* 16S rRNA and mosquito 18S rRNA genes was carried out as described previously [63]. *Wolbachia* counts were normalised against tick cell counts generated by the amplification of fragments of the tick *rpl6* gene as described previously [64]. All qPCR assays were run on a CFX Connect Real-Time PCR Detection System (Bio Rad, Watford, UK). A dilution series of synthetic oligonucleotides representing the full-length amplicons were used as standards for quantification by linear regression in CFX Manager software.

### 2.7. Sequence and Phylogenetic Analyses

Pair-end sequences obtained from PCR amplicons were assembled to produce a corrected consensus using 4Peaks (Nucleobytes B.V., Aalsmeer, The Netherlands) and Clustal Omega [65]. The sequences were then compared with published sequences using BLASTN against the non-redundant database at the National Center for Biotechnology Information (NCBI) GenBank and the *Wolbachia* MLST database on PubMLST [66]. The sequences were quality-trimmed manually and aligned with voucher sequences using Clustal Omega [65]. The phylogenetic relationship of the novel *Wolbachia* sp. isolated in this study with other existing *Wolbachia* sp. was inferred using the Bayesian Markov chain Monte Carlo in MrBayes 3.2 [67], with codon partitions, two runs of 3 million generations, four chains per run, sampling every 1000 trees generated and burn-in of 25% trees for each dataset. The best-fit model of nucleotide substitution was estimated by Akaike information criterion (AIC) as implemented in jModelTest 2.1.7 [68]. The models selected were GTR+G+I for 16S rRNA, GTR+G for concatenated genes of the *Wolbachia* MLST scheme [3], GTR+I for *coxA*, HKY+G for *fbpA* and GTR+G for the remaining genes.

## 3. Results

### 3.1. Propagation of Wolbachia Strains wStri and wAlb1 in Ixodes spp. Cell Lines

**Figure 1:**
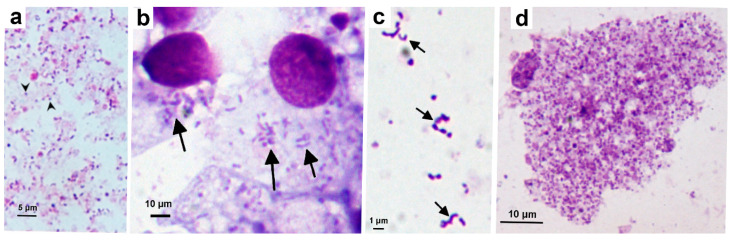
*Wolbachia* strain *w*Stri propagated in the *Ixodes scapularis* cell line ISE6. (**a**) Inoculum comprising cell-free *Wolbachia* (arrowheads) harvested from AeAl-2 mosquito cells; (**b**) ISE6 cells infected with *w*Stri (arrows) at passage 19; (**c**) Cell-free *Wolbachia* (arrows) harvested from infected ISE6 cells 2 weeks post infection; (**d**) Heavily-infected ISE6 cell, 4 weeks after passage of infected cells onto uninfected cell layer. Giemsa-stained cytocentrifuge smears.

Cell-free *Wolbachia* strain *w*Stri bacteria, harvested from heavily-infected AeAl-2 cells (Figure 1a), were initially used to infect ISE6, ISE18 and IRE11 cells. Once infection was established (Figure 1b), *w*Stri grew equally well in all three tick cell lines, and was routinely maintained in ISE6 cells by diluting infected cells with uninfected cells 1:10 or 1:20 every 10 to 20 days through 29 passages over a 14-month period. Cell-free *Wolbachia* harvested from infected ISE6 cells were pleomorphic, including round, rod- and crescent-shaped bacteria (Figure 1c). There was a pathological effect of *w*Stri on ISE6 cells (Figure 1d) and it was necessary to subculture infected cells onto fresh cell layers. Infected cells were hypertrophied and pyknotic.

Cell-free *Wolbachia* strain *w*AlbB bacteria, harvested from heavily-infected Aa23 cells, were initially used to infect ISE6, ISE18 and IRE11 cells. All three cell lines were successfully infected; *w*AlbB grew more slowly than *w*Stri, without causing any obvious cytopathic effects, and was routinely maintained in ISE6 cells by diluting infected cells with uninfected cells 1:5 every 4 weeks through 5 passages over a 5-month period.

### 3.2. Propagation of Wolbachia Strain wAlbB in a R. microplus Cell Line

**Figure 2:**
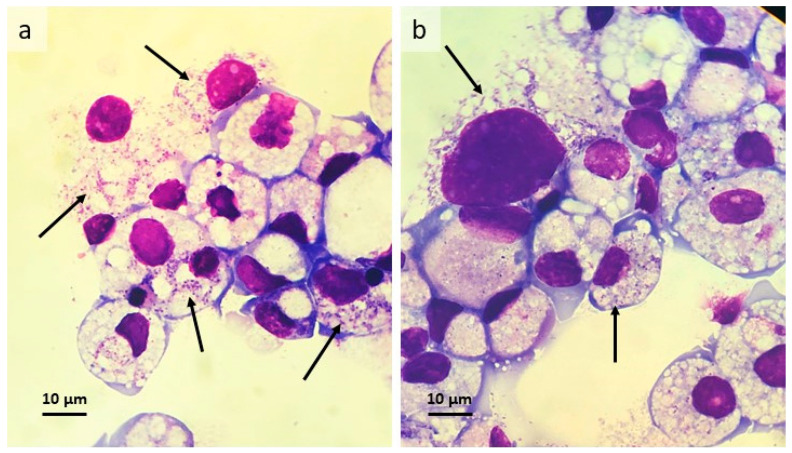
*Rhipicephalus microplus* cell line BME/CTVM23 infected with *Wolbachia* strain *w*AlbB at passage 2, 8 months after initial infection. (**a,b**) Giemsa-stained cytocentrifuge smears, arrows indicate heavily-infected cells.

A single BME/CTVM23 culture was inoculated with cell-free *w*AlbB bacteria harvested from C6/36 cells and incubated at 28 °C. A single C6/36 culture was inoculated at the same time as a positive control. Weekly inverted microscope examination did not reveal any obvious deleterious effects on the tick or mosquito cells. Intracellular bacteria were detected 2 weeks p.i. in both tick and mosquito cells; as expected, the C6/36 cells were heavily infected by week 4, while in BME/CTVM23 cells the bacteria gradually increased in both infection rate (from <1% to 5% cells infected) and infection level (from <10 to >50 bacteria per cell) over the subsequent 4 months. Then, during the following 6 weeks, the bacterial growth rate increased rapidly until around 50% of cells were infected with up to >100 bacteria visible per cell. At this point the first subculture onto fresh BME/CTVM23 cells was carried out; four weeks later this passage 1 culture was heavily infected and was passaged onto fresh BME/CTVM23 cells after a further 7 weeks. The passage 2 culture displayed a heavy infection between 8 and 12 weeks later (Figure 2a,b).

**Figure 3:**
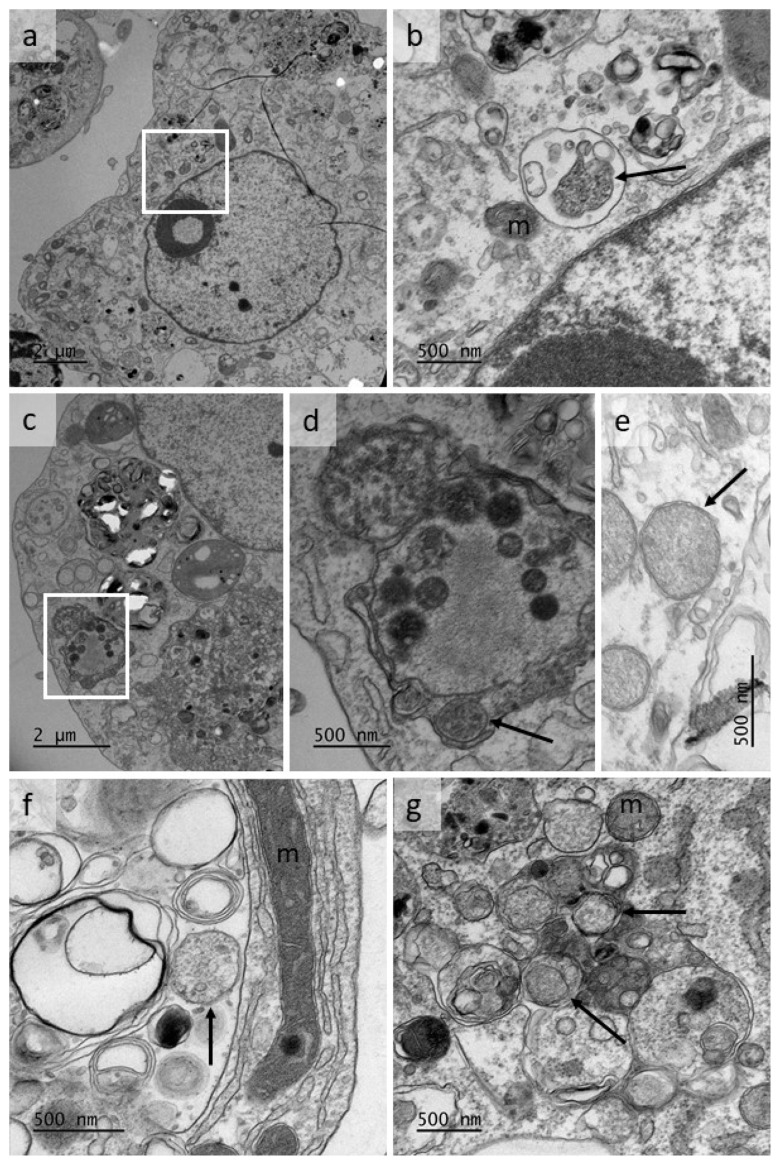
Transmission electron micrographs showing putative bacteria in the cytoplasm of cells of a culture of *the Rhipicephalus microplus* cell line BME/CTVM23 infected with *Wolbachia* strain *w*AlbB. The cells were processed from a passage 1 culture, 10 months after initial infection; m = mitochondrion. (**a**,**c**) low magnification views of infected cells; (**b**,**d**) enlarged areas of cells shown in a, c respectively, showing bacteria-like structures (arrows); (**e**,**f**,**g**) bacteria-like structures in BME/CTVM23 cell cytoplasm (arrows).

Transmission electron microscope examination of *w*AlbB-infected BME/CTVM23 cells at passage 1, 10 months after initial infection, revealed putative double membrane-bound bacteria resembling *Wolbachia* propagated in infected mosquito cell lines [56,69] and observed in the sand flea *Tunga penetrans* [14], the filarial nematode *Brugia malayi* [70] and the fruit fly *Drosophila melanogaster* [71]. Bacteria were seen either within membrane-bound compartments shared with a variety of structures including host-cell components (membranous whorls) and possible vesicles of bacterial origin, as reported previously [70] (Figure 3a–d,f,g), or apparently free in the cytoplasm (Figure 3e,g). These structures were easily distinguished from host cell mitochondria in which cristae were visible (Figure 3b,f,g).

Amplification by *Wolbachia* 16S rRNA qPCR confirmed the identity of the bacteria in the cultures and revealed a *w*AlbB infection level in the BME/CTVM23 cells of over 1000 bacteria per haploid host genome-equivalent. The failure to amplify by qPCR any product from the same sample using mosquito 18S rRNA primers confirmed the absence of contaminating C6/36 cells in the infected tick cell culture.

### 3.3. Isolation and Propagation of a Novel Wolbachia Strain from Malaysian Cat Fleas

**Figure 4:**
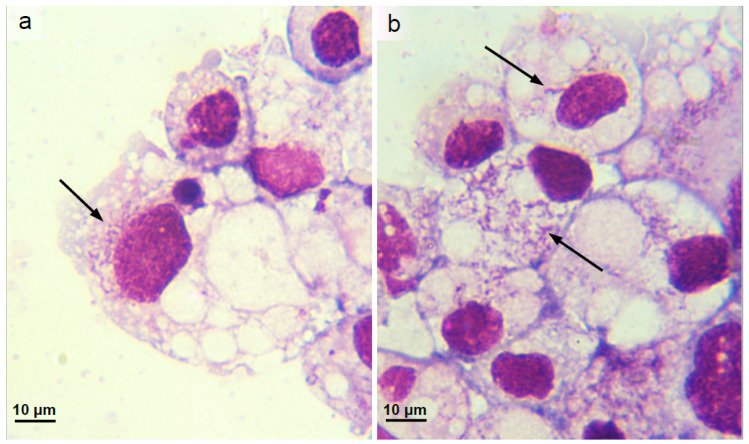
*Ixodes scapularis* cell line IDE8 infected with *Wolbachia* strain *w*CfeF isolated from Malaysian *Ctenocephalides* sp. cat fleas. (**a**,**b**) Parent culture, 8 months after inoculation with flea organs; Giemsa-stained cytocentrifuge smears, arrows indicate infected cells.

Seven pools of flea organs were inoculated into separate IDE8 cell cultures; of these, a single pool of five individual *Ctenocephalides* sp. female flea organs yielded a *Wolbachia* 16S rRNA and *wsp* PCR-positive culture. A fragment of the *Wolbachia wsp* gene was amplified from DNA extracted from the culture at three months p.i. and was still detectable by PCR at the time of writing (nine months p.i.). The *Wolbachia wsp* gene was also detectable by PCR in passages 1 and 2 at one month after subculture. The infected cells did not exhibit any cytopathic effects detectable by weekly inverted microscope examination. Intracellular bacteria were observed in Giemsa-stained cytocentrifuge smears prepared from the parent culture at eight months p.i., with approximately 25% of cells infected (Figure 4a,b). To further characterise the bacterium isolated from the Malaysian cat fleas, fragments of the *Wolbachia coxA*, *fbpA*, *ftsZ*, *hcpA* and *gatB* genes were also amplified from DNA extracted from the parent infected IDE8 cell culture at 5 months p.i. The resultant sequences from the novel *Wolbachia* isolate, designated *w*CfeF, were deposited in the NCBI GenBank database under accession numbers MT584103 (16S rRNA), MT577878 (*coxA*), MT577879 (*fbpA*), MT577880 (*ftsZ*), MT577881 (*gatB*) and MT577882 (*hcpA*), and compared with published sequences to determine its relationship with other *Wolbachia* strains.

**Figure 5:**
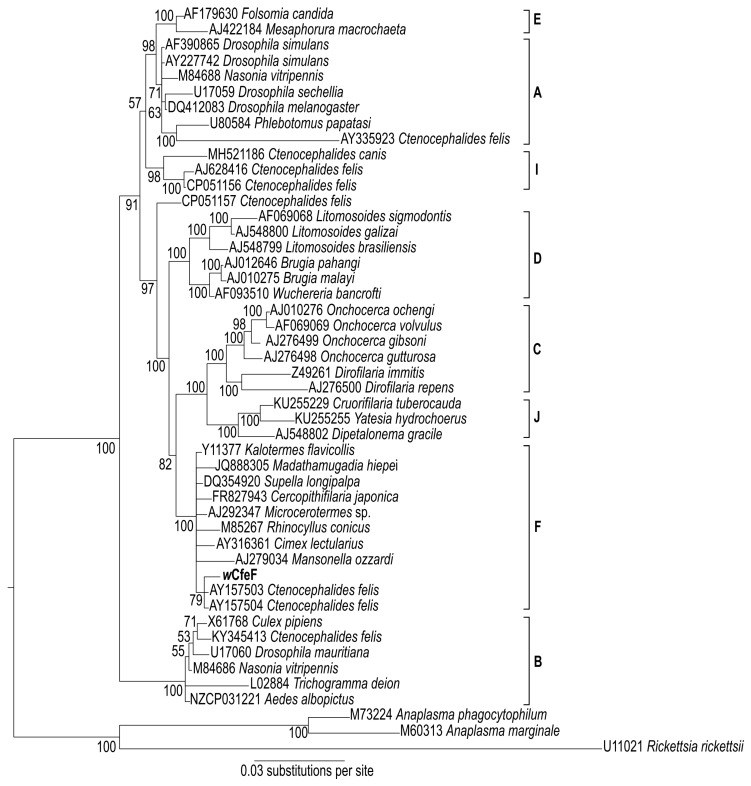
Bayesian inference phylogeny based on partial 16S rRNA sequences (1599 nucleotide positions) of novel *Wolbachia* strain *w*CfeF (**in bold**) isolated from Malaysian cat fleas, and of *Wolbachia* strains from the indicated hosts. Numbers at nodes represent Bayesian posterior probabilities (%). Accession numbers are given for the sequences in the NCBI GenBank database. Other members of the Rickettsiales, *Rickettsia rickettsii*, *Anaplasma phagocytophilum* and *Anaplasma marginale*, were used as an outgroup.

**Figure 6:**
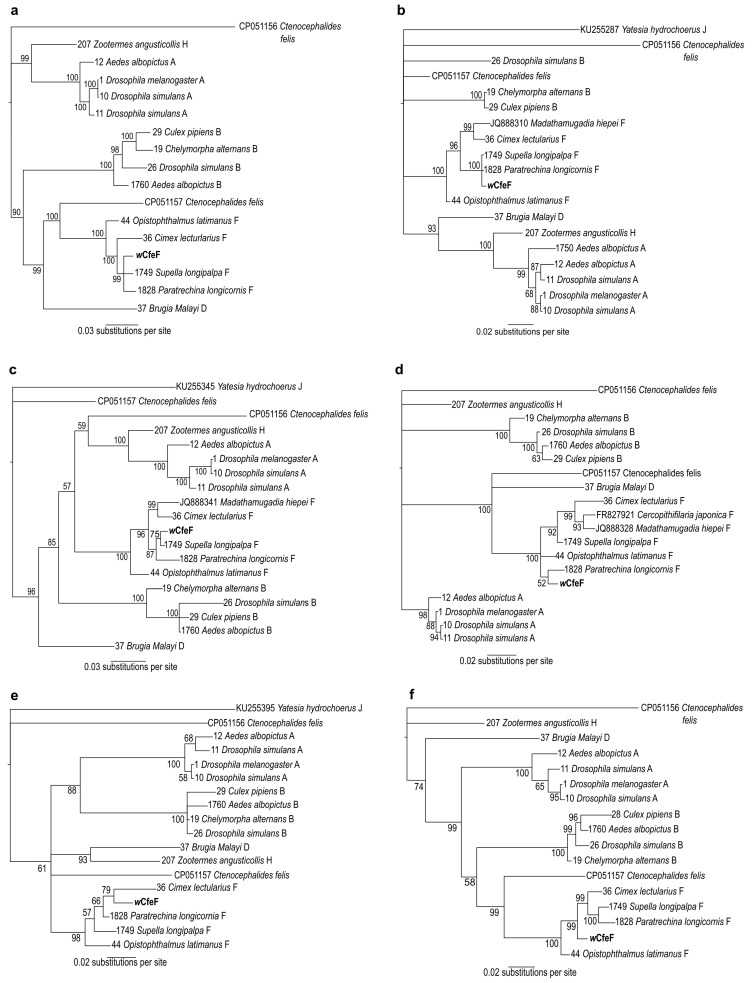
Bayesian inference phylogeny based on concatenated and individual MSLT gene sequences of novel *Wolbachia* strain *w*CfeF (**in bold**) isolated from Malaysian cat fleas, and of *Wolbachia* strains from the indicated hosts. (**a**) Concatenated MLST genes (2082 nucleotide positions); (**b**) *coxA*; (**c**) *fbpA*; (**d**) *ftsZ*; (**e**) *hcpA*; (**f**) *gatB*. Numbers at nodes represent Bayesian posterior probabilities (%). Sample ID and accession numbers are given respectively for the sequences from the *Wolbachia* PubMLST and NCBI GenBank databases.

A phylogenetic tree based on partial 16S rRNA sequences (Figure 5) positioned the *w*CfeF isolate within the clade consisting of arthropod and filarial *Wolbachia* strains from the F supergroup (posterior probability = 100). These include two *Wolbachia* strains previously identified in *Ctenocephalides felis* from Georgia, USA [15]. However, since only a short fragment of the 16S rRNA gene was used for phylogenetic construction, further investigation using additional gene sequences was necessary to determine its phylogenetic position. For this we used the concatenated and individual gene sets from the *Wolbachia* MLST scheme: fragments of the *coxA*, *fbpA*, *ftsZ*, *hcpA* and *gatB* genes [3]. The phylogeny of the concatenated MLST genes showed the clustering of *w*CfeF with *Wolbachia* strains from the F clade, including those from insects such as *Supella longipalpa* (Blattodea), *Paratrechina longicornis* (Hymenoptera) and *Cimex lectularius* (Hemiptera) (Figure 6a). Phylogenies based on individual genes showed similar positioning of *w*CfeF within the F clade (Figure 6b–f). In these phylogenies, the current isolate appeared to be distinct from the other *Wolbachia* strains from *C. felis* that fall within other clades [26]. Altogether, the findings here support the placement of the *w*CfeF isolate within the F supergroup.

## 4. Discussion

It is clear from our results that *Wolbachia* can invade and replicate in tick cells, at least in vitro. We demonstrated susceptibility of three cell lines derived from *I. scapularis* and one each from *I. ricinus* and *R. microplus*. Two laboratory-cultured strains and one novel field strain of *Wolbachia*, originating from diverse insect groups (mosquitoes, leafhoppers and fleas) and belonging to different supergroups (B and F), were able to infect and grow in tick cells. Special procedures, such as the shell vial technique or centrifugation, were not necessary for the initial infection of tick cells with *Wolbachia*, and infected cultures were maintained for long periods without the need for special incubation conditions. As seen in chronically-infected mosquito cell cultures [69,72], tick cell cultures infected with *Wolbachia* strains *w*AlbB and *w*CfeF did not display any cytopathic effect over prolonged periods in vitro. In contrast, *Ixodes* spp. cultures heavily infected with the *w*Stri strain displayed cytopathic effects, manifest as hypertrophied or pyknotic cells; the same strain in mosquito cells (AeAl-2) caused heavily infected cells to lose the ability to attach to a surface [56].

Considered together, all the tick cell lines used in the present study have previously been shown to be permissive for a wide range of intracellular bacteria transmitted and/or harboured by invertebrates. These include representatives of the genera *Anaplasma* [73,74], *Cardinium* [49,75], *Ehrlichia* [76,77,78], *Neoehrlichia* [79,80], *Mycobacterium* [81], *Rickettsia* [51,54,62,82,83,84] and *Spiroplasma* [62,85]. A previous study reported the propagation of a bacterium, then known as *Wolbachia persica*, in a *Dermacentor albipictus* tick cell line [86], but this bacterium was later found to belong to the genus *Francisella* [87]. Although the majority of these bacteria are tick-transmitted, exceptions include the predominantly flea-transmitted *Rickettsia felis* [83] and *Mycobacterium leprae*, the causative agent of leprosy, transmission of which may be associated with biting insects and ticks [81,88]. Tick cells similarly support the replication of a wide range of arboviruses transmitted not only by ticks but also by mosquitoes, sand flies and midges [89]. Therefore, it is not surprising that tick cell lines should be susceptible to infection with multiple strains of *Wolbachia* of insect origin. The ability of the novel Malaysian flea-derived *Wolbachia* isolate to infect and grow in a tick cell line confirmed the usefulness of tick cell lines in cultivating bacterial species of which ticks are not the natural hosts.

The cell lines used in the present study were also chosen because they belong to species in which *Wolbachia* has been detected by molecular techniques: *I. scapularis* [33,44], *I. ricinus* [34,35,36,38,43] and *R. microplus* [37,41]. Other tick species reported to harbour *Wolbachia* DNA include the ixodid ticks *Amblyomma americanum* [39], *Rhipicephalus sanguineus* [32], *Dermacentor silvarum* [45] and *Haemaphysalis hystricis* [40] and the argasid tick *Ornithodoros rietcorreai* [42]. None of these studies included a screen for insect DNA; concordance between the presence of *Wolbachia* and nematodes was not demonstrated [39]. To date, only two studies on *I. ricinus* [47,48] have attempted to make the connection between *Wolbachia* and parasitic insects by screening samples for the presence of DNA from both bacteria and the parasitic wasp *Ixodiphagus hookeri*.

The intracellular morphology of the *Wolbachia* strains, as revealed in Giemsa-stained smears, was quite similar to that of other members of the Rickettsiales propagated in tick cell lines, such as *Anaplasma marginale*, *Ehrlichia ruminantium*, *Neoehrlichia mikurensis* and *Rickettsia raoultii* [54,73,77,80]. Purple-staining, pleomorphic bacteria were located singly or in small groups in the cytoplasm; no bacteria were seen in cell nuclei. The ultrastructural morphology of the *w*AlbB strain in BME/CTVM23 cells was more difficult to interpret. Readily-identifiable bacteria, such as those seen in *R. raoultii*-infected cells of the same line [54], were not obvious. Where bacteria could be identified, they were generally situated within large, membrane-bound vacuoles also occupied by a variety of structures commonly seen in electron micrographs of tick cells (lipid membrane whorls and vesicles of varying size and shape). The origin of the vesicles is unclear; the presence of secretory vesicles in vacuoles occupied by the *w*MelPop strain of *Wolbachia* in *D. melanogaster* brain cells was reported previously [71], and the authors speculated that these could have originated from the endoplasmic reticulum. However, in a detailed ultrastructural study of *Wolbachia* in *B. malayi* nematodes, immunogold labelling was used to show that vesicles found in association with *Wolbachia* were of bacterial origin [70].

Although over a dozen *Wolbachia* strains (including *w*Stri, *w*AlbB and *w*MelPop) can be propagated continuously within insect cell culture systems, they belong exclusively to the more well-characterised A and B supergroups [69,72,90]. To the best of our knowledge, the new Malaysian *Wolbachia* strain is the only F supergroup member to be isolated into a cell line to date, the only flea-derived strain to be conclusively isolated and propagated over a prolonged period and the only *Wolbachia* field strain to be isolated into a tick cell line. A previous study did report the isolation of *Wolbachia*-like organisms from *C. felis* alongside *Rickettsia felis* in ISE6 cells; however, these putative *Wolbachia* were only identified morphologically and molecular confirmation was not presented [91].

The *Wolbachia* F supergroup is unique as members have been found in both arthropod and nematode hosts [92,93]. A previous study experimentally demonstrated a nutritional mutualism relationship between an F supergroup *Wolbachia* (*w*Cle) and its host, the bedbug *Cimex lectularius* [20]. To the best of our knowledge, the only phenotypic data pertaining to *Wolbachia* infection in a flea was a comparison between laboratory and wild populations of the gerbil flea, *Synosternus cleopatrae*, that detected a negative impact of infection on reproductive success in the laboratory colony but not in the wild population [94]. Interestingly, the recent completion of the *C. felis* genome from a laboratory colony in California led to the generation of two complete *Wolbachia* genomes, neither of which was placed in supergroup F [26]. One of these genomes, designated *w*CfeT, contains a complete biotin synthesis operon and may be involved in providing nutrients to the host cell. The second genome, *w*CfeJ, encodes a toxin-antidote system similar to that responsible for CI in dipteran hosts. However, experimental confirmation of mutualistic or parasitic roles for these *Wolbachia* in *C. felis* has not been published. The availability of a *Wolbachia* isolate from the F supergroup growing in a cell line will allow for further genetic and phenotypic characterisation at the cellular level, which could be useful in elucidating the *Wolbachia*-host relationship for this particular strain.

Despite the completion of the *C. felis* genome project and the detection of *Wolbachia* DNA in *Ctenocephalides* spp. fleas in several other studies [15,16,22,24,26], actual *Wolbachia* bacteria had not been visualised or isolated from fleas of this genus prior to the present study, although immunohistochemistry and transmission electron microscopy identified *Wolbachia* in the sand flea *T. penetrans* [14]. Sequence and phylogenetic analyses from separate studies suggest *Ctenocephalides* sp. fleas may not only host a wide range of *Wolbachia* strains from several distinct supergroups, but may also contain strains from a novel, divergent clade [15,16,22,26,93]. In Malaysia, DNA of *Wolbachia* from the B and F supergroups was detected in *C. felis* [22]; however, the sequences are not available in public databases for comparison with the novel *w*CfeF isolate. The phylogenies of 16S rRNA and the MLST scheme genes provided compelling evidence for the placement of the *w*CfeF isolate within the F supergroup, similar to the *Wolbachia* strains previously reported from North American *C. felis* [15].

Reports of *Wolbachia* from Malaysia and the wider Southeast Asian region have primarily concerned the A and B supergroup *Wolbachia* from mosquitoes, owing to national research priorities placed on mosquito-borne diseases [95,96,97]. A number of studies also investigated the presence of *Wolbachia* in other insects, such as the tephritid fruit flies and the butterfly *Hypolimnas bolina*, as well as filarial nematodes including *Brugia pahangi* and *Onchocerca borneensis* [98,99,100,101]. The present study represents the first isolation and genetic characterisation of a *Wolbachia* strain from *Ctenocephalides* sp. fleas in this region.

The phylogenies from the concatenated genes and a subset of individual MLST genes (*coxA*, *fbpA* and *ftsZ*) showed that *w*CfeF appeared to be closely related to *Wolbachia* strains from the longhorn crazy ant *P. longicornis*, and the brown-banded cockroach *S. longipalpa* [102,103]. Results from a recent study suggested the occurrence of horizontal transfer of F group *Wolbachia* between *Myrmecophilus* spp. ant crickets and their ant hosts, including *P. longicornis* [102]. Currently, the infection events contributing to the occurrence of multiple *Wolbachia* strains in *C. felis* are still not fully understood, although it has been suggested that the predation of fleas by other insect species could be a possible mode of transmission between distantly-related hosts [20].

It is important to consider the possibility of there being more than one *Wolbachia* strain within the *w*CfeF culture since the starting inoculum consisted of organs from five individual fleas. Examination of our sequencing trace files revealed a small number of double peaks at several nucleotide positions in some amplicon sequences, suggesting the possibility of strain variations. However, the phylogenies based on the individual genes indicate that, even if more than one strain was present, they must be closely related as the placement of the novel strain *w*CfeF within the F clade was consistent across all tested loci. Further studies, including whole genome sequencing, should be carried out to verify the phylogenetic relationship between *w*CfeF and other *Wolbachia* strains, to establish the host cell range capable of supporting its in vitro propagation, and to determine its relationship with such host cells. Moreover, the taxonomy and population genetics of *Ctenocephalides* spp. in South-East Asia is complex, with two “tropical clusters” of *C. felis* as well as *C. orientis* (previously *C. f. orientis*) being found in the region [104]. Due to the emphasis on bacterial isolation from fresh material in the current study, detailed morphometric or molecular characterisation of the fleas was not attempted, but it is important to note that *C. orientis* has a strong host preference for dogs and, to a lesser extent, small ruminants [104,105]. Therefore, further studies are needed to determine the possible range of flea species and subspecific clades harbouring *w*CfeF in Malaysia and the wider South-East Asian region.

## 5. Conclusions

Tick cell lines can now be added to panels of insect, predominantly mosquito, cell lines to enhance the capability to isolate field strains of *Wolbachia* from naturally-infected arthropods. The ability to propagate *Wolbachia* continuously in tick cell lines provides a platform to examine how these bacteria might modulate the replication of tick-borne arboviruses, bacteria and protozoa, potentially leading to novel control strategies for tick-borne diseases. In light of our demonstration, that tick cells are capable of supporting long-term infection with *Wolbachia*, the exact nature of the relationship between the bacterium, ticks and other parasitic arthropods such as *Ixodiphagus* in the field should be re-examined.
